# Are you laughing at me? Neural correlates of social intent attribution to auditory and visual laughter

**DOI:** 10.1002/hbm.24806

**Published:** 2019-10-22

**Authors:** Thomas Ethofer, Sophia Stegmaier, Katharina Koch, Maren Reinl, Benjamin Kreifelts, Lena Schwarz, Michael Erb, Klaus Scheffler, Dirk Wildgruber

**Affiliations:** ^1^ Department of General Psychiatry University of Tuebingen Tuebingen Germany; ^2^ Department of Biomedical Resonance University of Tuebingen Tuebingen Germany; ^3^ Max‐Planck‐Institute for Biological Cybernetics University of Tuebingen Tuebingen Germany

**Keywords:** anterior cingulate cortex, hostility, laughter, medial prefrontal cortex, social pain

## Abstract

Laughter is a multifaceted signal, which can convey social acceptance facilitating social bonding as well as social rejection inflicting social pain. In the current study, we addressed the neural correlates of social intent attribution to auditory or visual laughter within an fMRI study to identify brain areas showing linear increases of activation with social intent ratings. Negative social intent attributions were associated with activation increases within the medial prefrontal cortex/anterior cingulate cortex (mPFC/ACC). Interestingly, negative social intent attributions of auditory laughter were represented more rostral than visual laughter within this area. Our findings corroborate the role of the mPFC/ACC as key node for processing “social pain” with distinct modality‐specific subregions. Other brain areas that showed an increase of activation included bilateral inferior frontal gyrus and right superior/middle temporal gyrus (STG/MTG) for visually presented laughter and bilateral STG for auditory presented laughter with no overlap across modalities. Similarly, positive social intent attributions were linked to hemodynamic responses within the right inferior parietal lobe and right middle frontal gyrus, but there was no overlap of activity for visual and auditory laughter. Our findings demonstrate that social intent attribution to auditory and visual laughter is located in neighboring, but spatially distinct neural structures.

## 1 INTRODUCTION

Laughter represents a strong social signal. This is impressively documented by the fact that the probability of its occurrence is 30 times higher in the presence of others as compared to situations without other humans being present (Provine, 2000). Charles Darwin hypothesized that the evolutionary basis of laughter was its function as a social expression of happiness, and that this rendered a cohesive survival advantage to the group (Darwin, 1872). However, it should be noticed that laughter is a multifaceted social signal, which goes beyond the social bonding, but can also serve as a social rejection cue (Alter & Wildgruber, 2019; Eibl‐Eibesfeldt, 1970; Papousek et al., 2014). Taunting laughter, for example, aims at humiliating and socially excluding the recipient from a group. Thus, these laughter types express opposite intentions: social acceptance as opposed to social rejection. Tickling laughter, on the other hand, is a reflexive behavior to somatosensory stimulation (Ruch & Ekman, 2001), which is also emitted by nonhuman primates (Meyer, Baumann, Wildgruber, & Alter, 2007; van Hooff, 1972) and one of the first laughter types expressed by children (Washburn, 1929). While it is argued to play an evolutionary role in social play and bonding (Provine, 2004), the experience of being tickled has a “tipping point” with a reported change of experience from pleasantness to unpleasantness or even social aversion (Alter & Wildgruber, 2019). Consequently, tickling can be associated with defensive behavior and lead to increased activation of brain regions involved in pain perception as well as fight and flight responses (Wattendorf et al., 2013). Due to these contrary responses, tickling laughter is seen as ambiguous social stimulus.

In the current study, we presented visual or acoustic stimuli of friendly, tickling, and taunting laughter and instructed the participants to rate the perceived social intent on a four‐point scale during functional magnetic resonance imaging (fMRI). A previous study using audiovisual laughter as stimuli demonstrated that neural responses within the right superior temporal cortex increase with perceived negative social intent in children and adolescents (Martinelli et al., 2019). Moreover, numerous fMRI studies have been conducted to identify the neural correlates of social rejection/exclusion. The majority of these studies relied on the so‐called Cyberball task (Williams & Jarvis, 2006) involving exclusion from a ball‐tossing game by virtual players, which the participant is made to believe are real individuals. Other paradigms employed to investigate social rejection are the Social Judgment and Chatroom tasks during which participants evaluate others based on photographs and receive feedback on whether the other persons are interested in them (e.g., Somerville, Heatherton, & Kelley, 2006) as well as the virtual handshake task where others accept or reject the participant's handshake (Lee et al., 2014). A meta‐analysis of neuroimaging studies revealed the medial prefrontal cortex (mPFC) extending into the anterior cingulate cortex (ACC) and a cluster in the posterior cingulate cortex extending into the precuneus (PCC/PC) as well as the inferior frontal gyrus (IFG) as neural correlates of “social pain” induced by social rejection/exclusion (Vijayakumar, Cheng, & Pfeifer, 2017).

The neural correlates of perceived social acceptance have received much less attention than those of social rejection in the past. Only one neuroimaging study in healthy participants explicitly addressed effects due to social acceptance revealing that previous experience of positive social encounters can enhance activation to stimuli of biological motion within the right supramarginal gyrus and posterior superior temporal sulcus (Bolling, Pelphrey, & Kaiser, 2013). Furthermore, some studies using the Cyberball paradigm also descriptively reported the activation maps comparing the fair play versus social exclusion conditions. These maps revealed stronger activation in lateral frontoparietal cortices which occurred more consistently within the right than left hemisphere if the participant received the ball with an equal probability compared to the other players (fair play) than during social exclusion (Bolling et al., 2011a; Bolling et al., 2011b; Bolling et al., 2011c; Bolling, Pelphrey, & Vander Wyk, 2012; Bolling, Pelphrey, & Vander Wyk, 2015).

While previously employed paradigms relied on explicit and rather drastic signals (e.g., rejection/acceptance of a handshake or overt expression of sympathy/dislike based on a photograph), social intents are often expressed in a more implicit and subtle manner by nonverbal signals. It has long been recognized that laughter represents a nonverbal signal that is well suited to express messages ranging from sexual solicitation to aversion (Grammer, 1990). To investigate which brain areas are responsive to the social intentions expressed by laughter, we performed regression analyses addressing linear relationships between brain activation and individual ratings of social intent attribution. Based on previous results, we hypothesized that the activation within the right superior temporal cortex (Martinelli et al., 2019) as well as regions previously associated with processing social pain (i.e., mPFC/ACC, PCC/PC, and IFG; Vijayakumar et al., 2017) increase with negative social intent attribution, indicating perceived social rejection while activation in lateral frontoparietal areas increases if laughter is perceived as reflecting positive social intent, indicating a socially accepting attitude (Bolling et al., 2011a; Bolling et al., 2011b; Bolling et al., 2011c; Bolling et al., 2012; Bolling et al., 2015). Finally, we examined whether the neural representations of social rejection/acceptance expressed by auditory or visual laughter are situated in distinct or common brain areas.

## 2 METHODS

### 2.1 Participants

All demographic and psychometric data are given in mean ± *SD* and range. Fifty two healthy subjects (27 women, 29.2 ± 9.5 years; 19–57 years) participated in the study. Participants were recruited via public announcements and screened beforehand for any current and/or history of neurological and/or psychopathological impairments by trained psychologists using the SCID I and SCID II interviews (First, Spitzer, Gibbon, & Williams, 1995; First, Spitzer, Gibbon, & Williams, 1997). All participants were right‐handed as determined by the Edinburgh Handedness Inventory (Oldfield, 1971). Verbal intelligence of the participants (107 ± 15; range: 78–145) was assessed using a multiple‐choice vocabulary test (Mehrfachwortschatztest, MWT‐B, Lehrl, 1997). External assessment of affective symptoms based on the Hamilton Depression Rating Scale (Hamilton, 1960) did not reveal major affective symptoms in any of the study participants (0.5 ± 0.7; range: 0–2). Similarly, self‐assessment using the Beck Depression Inventory (Beck, Guth, Steer, & Ball, 1997) was not indicative for depressive symptoms (1.3 ± 1.6; range: 0–7). The study conformed to the code of Ethics of the World Medical Association (Declaration of Helsinki) and the study protocol was approved by the ethics committee of the medical faculty of the Eberhard‐Karls‐University Tübingen. All participants gave written informed consent to the study prior to participating. All neuroimaging and behavioral data acquired in this study are available on reasonable scientific request from the authors.

### 2.2 Stimulus material

To obtain laughter stimuli that vary across the dimension of social intention, we invited eight professional actors who portrayed friendly laughter, tickling laughter, and taunting laughter using a script‐based auto‐induction technique. During production of the video sequences, actors were wearing black head caps in front of a black background in order to minimize the influence of visual cues, which are not part of the human face. The video sequences were post‐processed to ensure equal duration (1.5 s) and quality using Adobe Premiere Pro CS3 software including editing of videos with respect to the alignment of the vertical facial symmetry axis and the size of the portrayed faces. Normalization of sound intensity to a mean of 70 dB was achieved by using PRAAT, version 5.1.07 (Boersma, 2001). From the generated stimulus material of 187 video sequences, 20 stimuli were selected for each laughter type which were balanced for gender of the actors (11 and 9 stimuli of female and male actors for each laughter type, respectively, for a more detailed description of the stimulus material, see Kreifelts et al., 2017; Kreifelts et al., 2014; Ritter et al., 2015).

The social intention of these stimuli was rated by 14 healthy subjects (7 females, mean age: 25.8 ± 3.3 years) in a prestudy to evaluate the impact of the communicational channel on the perceived intention. To this end, participants were asked to judge to which extent the laughter sequences express a positive emotional attitude toward the receiver signaling the intention to further positive social interaction and bonding (the employed German word for such socially inclusive laughter was “Anlachen”) or a negative emotional attitude toward the receiver signaling the intention to reduce positive social interaction and bonding (the employed German word for such socially excluding laughter was “Auslachen”). Thus, they were instructed to rate the social intent of the expressed laughter on a four‐point scale (−−, −, +, and ++) as strong/slight positive social intent (i.e., “Anlachen”) or strong/slight negative social intent (i.e., “Auslachen”). We chose the words “Anlachen” versus “Auslachen” instead of “Soziale Akzeptanz” (social acceptance) versus “Soziale Ablehnung” (social rejection) for the employed scale as the first are more frequently used in daily language whereas the latter represent technical terms that are more appropriate for academic use.

Percentages of positive social intent (strong or slight) and negative social intent (strong or slight) ratings were similar for stimuli presented as auditory (A) sound clips (positive: 45.0 ± 3.7%; negative: 55.0 ± 3.7%) and audiovisual (AV) movie clips (positive: 42.2 ± 3.1%; negative: 57.8 ± 3.1%) with no significant difference between modalities (paired T(19) = 1.34, *p* = .20, two‐tailed). Stimuli presented visually (V) as mute video clip were rated more frequently as representing positive social intent (positive: 58.5 ± 3.7%; negative: 41.5 ± 3.7%) than A or AV stimuli (both paired T(19) > 4.1, *p* < .001, two‐tailed).

### 2.3 Experimental design

In the fMRI experiment, we employed only A and V stimuli of the prestudy, but not the AV stimuli from which both classes of stimuli were generated to avoid crossmodal effects that might be induced by presentation of bimodal stimuli preceding unimodal stimuli as well as order effects that would result if AV stimuli were always presented following the unimodal stimuli. About 60 V and 60 A stimuli were presented using an event‐related design with an inter stimulus interval (ISI) ranging from 9 to 12 s (mean ISI = 10.5 s, jittered in steps of TR/4) during two consecutive fMRI sessions (30 V and 30 A stimuli per session). In addition, six null events with a duration of 10.5 s were randomly inserted (1 null event per 10 trials on average). The study participants were not informed that the auditory and visual stimuli were created from the same stimulus set and it was emphasized that there are no “correct” or “incorrect” responses, but that their individual perception of the laughter stimuli is important. They were instructed to rate the social intent of the expressed laughter on the same four‐point scale used in the prestudy (see above). Visual stimuli and the rating scale were presented via back‐projection onto a screen approximately 2 m behind the participants' head and viewed over a mirror mounted on the head coil. Auditory stimuli were presented via MRI‐compatible audio headphones. Stimulus presentation and recording of behavioral responses were carried out using the software Presentation (Neurobehavioral Systems, http://www.neurobs.com/). The acquisition of behavioral data was achieved using an MRI‐compatible response system for four fingers (Celeritas Fiber Optic Button Response System, Psychology Software Tools).

### 2.4 Neuroimaging data acquisition

High resolution structural T1‐weighted images (TR = 2.3 s, TE = 4.16 ms, TI = 0.9 s, voxel size: 1 × 1 × 1 mm^3^) and functional images (72 slices, slice thickness 2 mm + 1 mm gap, TR = 1.5 s, TE = 34 ms, voxel size: 2 × 2× 2 mm^3^, multi‐band acceleration factor 3) were collected with a 3 T scanner (PRISMA, Siemens, Erlangen, Germany) using a 20 channel head coil. Time series consisted of 478 images for each of the two sessions. For image distortion correction, a field map (36 slices, slice thickness 3 mm + 1 gap, TR = 0.4 s, TE(1) = 5.19 ms, TE(2) = 7.65 ms, voxel size: 3 × 3 × 3 mm^3^) was obtained.

### 2.5 Analysis of behavioral data

Only behavioral responses occurring within 5 s after stimulus onset were included in the analysis. The percentage for each of the four response options was calculated separately for visual and auditory trials and statistically compared using paired two‐sided *t*‐tests. As for two of the four statistical comparisons, the Kolmogorov–Smirnov test indicated that the assumption of normal distribution of the data is violated (*p* < .05), we additionally compared the data based on non‐parametrical testing (Wilcoxon test).

### 2.6 Analysis of fMRI data

Images were analyzed with statistical parametric mapping software (SPM12, Wellcome Trust Center for Neuroimaging, UCL, London, UK). Preprocessing comprised slice time correction, realignment, unwarping to correct for field distortions and to remove residual movement‐related variance due to interactions between motion and field distortions (Andersson, Hutton, Ashburner, Turner, & Friston, 2001), normalization to MNI space (Montreal Neurological Institute, resampled voxel size: 2 × 2 × 2 mm^3^) based on the unified segmentation approach integrated in SPM (Ashburner & Friston, 2005) and smoothing with a Gaussian filter (6 mm full width at half maximum). The first five functional images of each session were discarded from analysis to exclude measurements preceding T1 equilibrium. Statistical analysis relied on a general linear model with separate regressors for each of the 120 events using a stick‐function time locked to the onset of stimulus presentation and convolved with the hemodynamic response function. To remove low‐frequency components, a high‐pass filter with a cutoff frequency of 1/128 Hz was used. Serial autocorrelations were accounted for by modeling the error term as a first‐order autoregressive process with a coefficient of 0.2 (Friston et al., 2002) plus a white noise component (Purdon & Weisskoff, 1998). Data from first‐level general linear models were submitted to simple regression analyses based on individual behavioral responses and corresponding brain activation for each trial (i.e., beta images). Trials with missed responses were excluded from the simple regression analyses. The individual slopes estimated by these simple regression analyses were submitted to a second‐level random effects analysis. To detect differences across modalities, these slopes were contrasted for all brain areas identified by the simple regression analysis. To detect commonalities across modalities, conjunction analyses based on the conjunction null hypothesis (corresponding to a logical AND, Nichols, Brett, Andersson, Wager, & Poline, 2005) were used to examine whether activation clusters showing a linear relationship between behavioral responses and brain activation overlap for auditory and visually presented laughter (i.e., linear relationship between activation to auditory laughter and rating scores ∩ linear relationship between activation to visual laughter and rating scores). Assignment of anatomical structures to activation clusters relied on the automatic anatomical labeling tool integrated in SPM (Tzourio‐Mazoyer et al., 2002). All activations are reported using an uncorrected height threshold of *p* < .001. Correction for multiple comparisons was carried out at cluster level using an extent threshold of *k* ≥ 110 voxels (*p* < .05, family‐wise error [FWE] corrected). Average responses (mean ± *SE*) for each of the four response options within brain regions showing significant effects were calculated to visualize the effect of perceived social intent of laughter on corresponding brain activation.

## 3 RESULTS

### 3.1 Behavioral data

**Figure 1:**
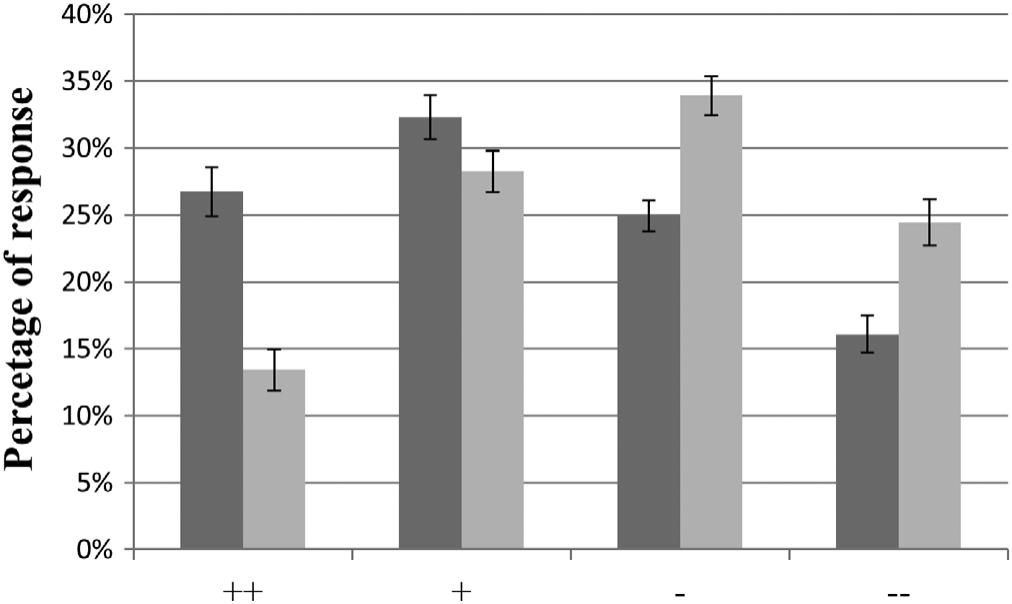
Behavioral data: Percentages of responses (mean ± *SE*) for strongly positive (++), slightly positive (+), slightly negative (−), strongly negative (−−) social intent attributions of visual (dark gray) and acoustic (light gray) laughter stimuli

Participants responded to 96.3% ± 1.0% of the presented trials. Although the visual and auditory stimuli were created from the same laughter sequences, higher percentages of positive social intent attributions (and correspondingly, lower percentages of negative social intent attributions) were found for laughter presented in the visual than auditory modality which is in agreement with the evaluations of the stimuli in the prestudy. The differences between auditory and visual trials were significant for all four response options as indicated by parametric (all paired T(51) > 2.45; all *p* < .01, two‐tailed) and non‐parametric (all Wilcoxon *Z* > 2.05, all *p* < .05, two‐tailed) tests. The distribution of the four different response options is presented in Figure 1.

### 3.2 fMRI data

**Figure 2:**
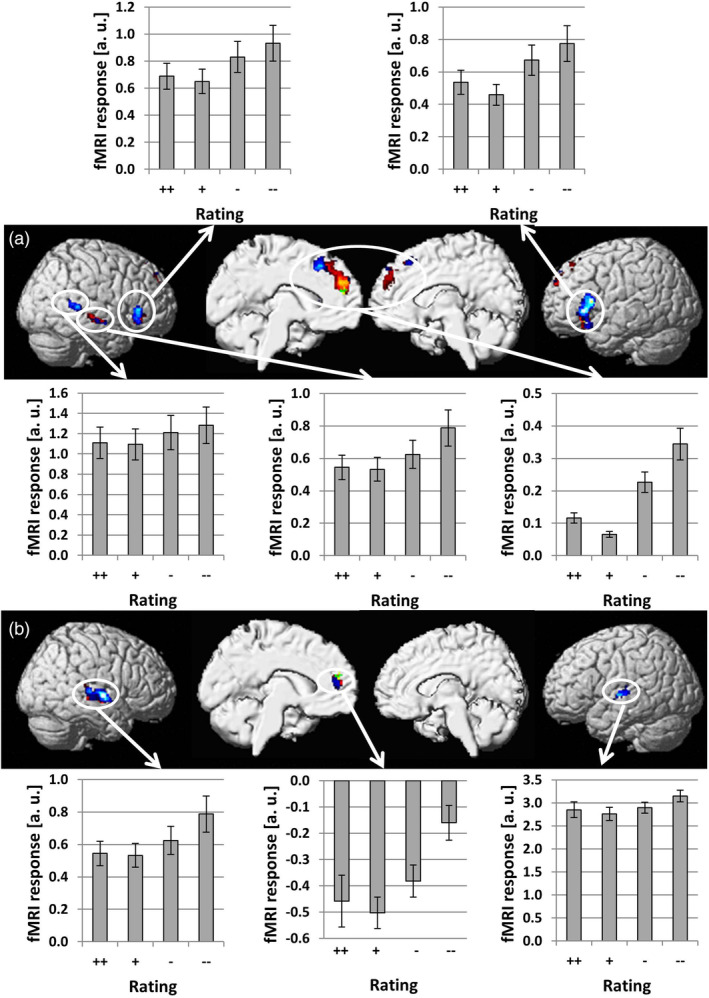
Brain areas showing a linear increase of hemodynamic responses with negative social intent attributions (social rejection) during perception of visual (a) and acoustic (b) laughter stimuli. Brain areas significant after correction for multiple comparisons (*p* < .05, FWE corrected) are shown in red/yellow if no significant differences across modalities were found and in blue/light blue if the regression slopes determined for the two modalities were significantly different [i.e., significantly stronger for visual than auditory laughter in (a) and significantly stronger for auditory than visual laughter in (b)]. A small area (green/light green) within the medial SFG was identified by the conjunction analysis [Color figure can be viewed at http://wileyonlinelibrary.com]

**Figure 3:**
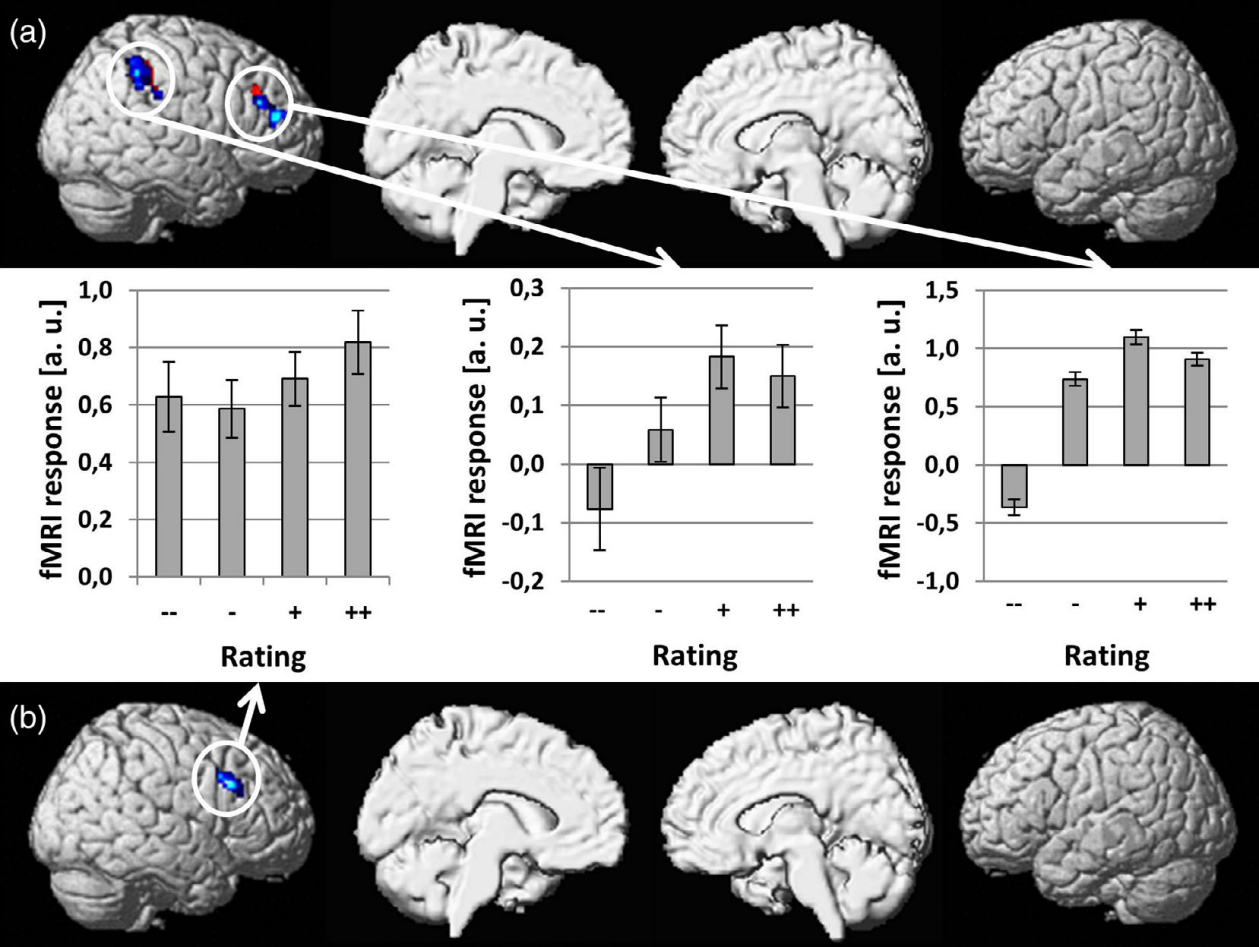
Brain areas showing a linear increase of hemodynamic responses with positive social intent attributions (social acceptance) during perception of visual (a) and acoustic (b) laughter stimuli. Brain areas significant after correction for multiple comparisons (*p* < .05, FWE corrected) are shown in red/yellow if no significant differences across modalities were found and in blue/light blue if the regression slopes determined for the two modalities were significantly different [Color figure can be viewed at http://wileyonlinelibrary.com]

We conducted simple regression analyses between brain activation and behavioral responses separately for visual and auditory laughter stimuli. Brain areas significant after correction for multiple comparisons (*p* < .05, FWE corrected) are shown in red/yellow if no significant differences across modalities were found and in blue/light blue if the regression slopes determined for the two modalities were significantly different (see Figure 2). In agreement with our a priori hypothesis, activation within the mPFC increased with negative rating of social intent for both visual and auditory laughter. These activation clusters were mostly situated within the medial superior frontal gyrus (SFG), but also extended into the ACC. The conjunction analysis revealed a small area within the medial SFG that showed overlap (MNI coordinates: *x* = −4; *y* = 50; *z* = 20; *Z* score = 3.34; cluster size = 15 voxels) for visually and auditory presented laughter (see green/light green area in Figure 2). Within this small area, no significant differences across modalities were found. Other brain areas that showed an increase of activation with negative social intent attributions included bilateral IFG, right mid and posterior superior/middle temporal gyrus (STG/MTG) for visually presented laughter and bilateral STG for auditory presented laughter (see Table 1). Increases of brain activation with positive social intent attributions were found in the right inferior parietal lobe (IPL) and right middle frontal gyrus (MFG) for visually presented laughter and in the right MFG for auditory presented laughter (see red/yellow areas in Figure 3 and Table 1) with no overlap across sensory modalities in the conjunction analysis.

**Table 1:** Brain regions identified by simple regression analysis between activation and social intent expressed by visual or auditory laughter

Brain region	MNI coordinates [*x*, *y*, *z*]	*Z* score	Cluster size
*Regression between negative social intent expressed by visual laughter and activation*			
Bilateral medial SFG	−4, −48, 30	5.38	672
Left IFG	−34, 20, −16	5.20	906
Right posterior STG/MTG	48, −38, 4	5.08	170
Right mid STG/MTG	54, −16, −8	4.82	130
Right IFG	56, 28, −2	4.26	222
*Regression between negative social intent expressed by auditory laughter and activation*			
Right mid STG	54, −10, 2	4.91	398
Left medial SFG	−6, 50, 14	4.80	129
Left mid STG	−48, −18, 2	4.02	116
*Regression between positive social intent expressed by visual laughter and activation*			
Right IPL	52, −44, 54	4.39	333
Right MFG	28, 32, 26	4.04	223
*Regression between positive social intent expressed by auditory laughter and activation*			
Right MFG	40, 16, 34	3.93	176

Abbreviations: IFG, inferior frontal gyrus, IPL, inferior parietal lobe, MFG, middle frontal gyrus, MTG, middle temporal gyrus, SFG, superior frontal gyrus, STG, superior temporal gyrus.

## 4 DISCUSSION

The current study aimed to delineate the neural correlates for processing laughter regarding its perceived social intent (i.e., social acceptance vs. social rejection) within the framework of an event‐related fMRI study capturing linear relationships between brain activation and individual ratings. Importantly, our study design allowed us to examine such effects separately for auditory and visual laughter.

Behavioral ratings revealed that participants judged visual stimuli with a significantly higher probability as socially accepting (and correspondingly with a lower probability as socially rejecting) than auditory stimuli. These findings obtained during fMRI were in agreement with results obtained in a prestudy outside the scanner. Furthermore, we also included AV stimuli in the prestudy serving as a reference of social intent attribution when the full information in voice and face is available. Social intent attribution of auditory and AV stimuli were highly similar suggesting that purely visual laughter stimuli elicit a positivity bias (i.e., a laughing face can be misjudged as expressing a positive social intent if the concomitant auditory stimulus cannot be perceived). While this bias resulted in more positive social intent attributions (and correspondingly less negative social intent attributions) in the visual than in the auditory modality, the overall distribution of responses across the four response options was sufficiently even enough to enable investigation of parametric relationships between brain activation and individual ratings. As explained above, we relied on a scale for rating of the social intent expressed by laughter (i.e., “Anlachen” vs. “Auslachen”) instead of the more technical terms social acceptance versus social rejection. This represents a limitation regarding the interpretation of our results within the framework of the previous literature on social acceptance/rejection, but we feel that both terms can be used interchangeably at the semantic level regarding the denomination of social intent of laughter and the neurobiological results of our study revealing similar brain activations as in previous studies on social rejection/acceptance support this view (see below).

So far, the vast majority of fMRI studies conducted to study the neural correlates of social rejection are based on the Cyberball paradigm (Williams & Jarvis, 2006) revealing the mPFC/ACC, PCC/PC, and IFG as key nodes for social pain induced by exclusion in such virtual game conditions (for a meta‐analysis, see Vijayakumar et al., 2017). The mPFC/ACC was also found to be hyperactive during social rejection within the framework of the Chatroom Task (Somerville et al., 2006) indicating that this area is sensitive to negative social feedback across various situations. The findings from our current study are compatible with this role of the mPFC/ACC as activity within this region increased with ratings signaling social rejection for both auditory and visual laughter. While there was some overlap of activation for the clusters identified for auditory and visual laughter, the larger parts of these two clusters (>88% of the auditory cluster and >97% of the visual cluster showed no overlap with the respective other cluster) were spatially distinct from each other with the auditory cluster situated more ventral than the visual cluster. The finding of spatially distinct neural structures was additionally supported by significant activation differences across modalities within these clusters. While these results argue for rather separate representations of social rejection expressed by visual and auditory laughter, the small overlapping part might still represent a convergence zone and could thus be a candidate region in future studies addressing AV integration of social laughter.

In contrast to previous studies based on the Cyberball task, no significant increase of activity with rating of social rejection expressed by auditory or visual laughter was found for the PCC/PC. A possible explanation for this finding could be the fact that the social exclusion condition in the Cyberball task signals rejection from more than one (in most cases two) individuals, while each laughter stimulus expressed feedback from exactly one individual. A particular sensitivity of the PCC/PC region to rejection from social groups could explain why no significant activation was found in our study or experiments relying on the Chatroom paradigm (Somerville et al., 2006), which also conveys feedback from exactly one person for a given stimulus. However, there are also other explanations for this difference in activation patterns across designs as social rejection in Cyberball tasks, but not the Chatroom task or our design using laughter stimuli, may also result in inhibition of motor preparation as well as perceptual expectancy violation due to not receiving the ball. Another region identified in a meta‐analysis on the neural correlates of social pain is the IFG adjacent to the orbitofrontal cortex (Vijayakumar et al., 2017). In our study, the activation of this area increased with individual ratings of social rejection for visual, but not auditory laughter indicating that processing of social information conveyed by laughter within this region is dependent on the sensory modality.

Moreover, brain activity increased with negative social intent attributions in distinct areas within the temporal lobe, but showed no overlap for auditory and visual laughter. For auditory laughter, these clusters were located in the middle part of the STG replicating previous results on representation of socially relevant information expressed by nonverbal social acoustic signals expressed by prosody (Ethofer et al., 2007; Ethofer, Van De Ville, Scherer, & Vuilleumier, 2009; Grandjean et al., 2005) and particularly the effect of negative social intent attribution expressed by AV laughter stimuli examined in a group of healthy children and adolescents (Martinelli et al., 2019). For visual laughter, two clusters along the middle and posterior part of the right superior temporal sulcus were found. The posterior superior temporal sulcus was also found active during social rejection as examined in the virtual handshake paradigm (Lee et al., 2014), which might reflect its role in integrating social meaning from various sources of biological motion including facial expressions (Said, Moore, Engell, Todorov, & Haxby, 2010), eye gaze (Ethofer, Gschwind, & Vuilleumier, 2011), or body postures (Basil, Westwater, Wiener, & Thompson, 2017) with their compatibility to social norms (Bahnemann, Dziobek, Prehn, Wolf, & Heekeren, 2010) while the activation along the middle part of the superior temporal sulcus could be driven by lip reading (Calvert et al., 1997).

Interestingly, inspection of beta estimates of the four response options revealed for all brain areas that activation and negative social intent attributions did not follow a pure linear relationship. This was due to the fact that the response option strongly positive (++) consistently resulted in stronger activation than slightly positive (+) which was always the response option yielding the lowest activation level. A possible explanation for this response pattern might be that slightly positive stimuli grab the lowest level of attention as they are more often encountered in real life social situations and thus perceived as a kind of “social default.” While we cannot make strong inferences on this issue as direct comparisons between the beta estimates of the four response options failed to reach significance, this observation might still serve as a starting point for future studies to inform hypotheses on such nonlinear relationships between brain activation and rating of socially relevant signals.

So far, there is much more knowledge on the neural correlates of social rejection than social acceptance, which is most probably due to the fact that social rejection has a stronger reference to symptoms experienced in clinical populations. In social anxiety, fears concerning negative evaluation and social rejection represent even the core symptom of the disorder (Trower & Gilbert, 1989). Altered processing of social acceptance, however, has been recently described for adolescent (Brown et al., 2017) and adult patients (Malejko et al., 2018) with borderline personality disorder calling for a better characterization of the neural correlates of social acceptance also in healthy participants. The current limitation of knowledge is not due to a lack of data, but a lack of analyses tackling this scientific question as most researchers who employ the Cyberball paradigm exclusively report activations to social exclusion versus fair play, but not the reverse contrast. Only one group also reported results on social acceptance (i.e., fair play > social exclusion, Bolling et al., 2012; Bolling et al., 2015; Bolling et al., 2011a; Bolling et al., 2011b; Bolling et al., 2011c) demonstrating activations in lateral frontoparietal areas. The findings of our study showing that activation in right IPL and MFG increase with ratings of social inclusion of laughter stimuli are compatible with these findings obtained in Cyberball paradigms. These converging findings suggest a more general role of the right IPL and MFG in capturing situations in which the individual is part of a group or approached with socially accepting signals.

In conclusion, the current study allowed us to disambiguate brain structures for processing perceived positive versus negative social intent expressed by laughter. Negative social intent attribution recruited the mPFC/ACC for both visual and auditory laughter, which nicely dovetails with results from neuroimaging studies relying on the Cyberball paradigm to induce feelings of social rejection. Interestingly, there was little overlap in activation between sensory modalities with auditory laughter being represented more rostral than visual laughter within the mPFC/ACC. Similarly, perceived positive social intent expressed by laughter recruited the right IPL and right MFG with no overlap across modalities. These findings argue for distinct rather than common processing of social signals conveyed by visual and acoustic laughter stimuli.

## CONFLICT OF INTEREST

The authors declare no potential conflict of interest.

## Data Availability

The data that support the findings of this study are available on request from the corresponding author. The data are not publicly available due to privacy or ethical restrictions.
